# Neutrophil to lymphocyte ratio as a predictor for treatment of radiation‐induced brain necrosis with bevacizumab in nasopharyngeal carcinoma patients

**DOI:** 10.1002/ctm2.583

**Published:** 2022-01-24

**Authors:** Jinhua Cai, Ruiqi Xue, Zongwei Yue, Zhan Zhang, Lei He, Honghong Li, Yi Li, Xiaoming Rong, Xiaoni Zhang, Yongteng Xu, Yamei Tang

**Affiliations:** ^1^ Department of Neurology Sun Yat‐sen Memorial Hospital Sun Yat‐sen University Guangzhou People's Republic of China; ^2^ Guangdong Provincial Key Laboratory of Malignant Tumor Epigenetics and Gene Regulation Sun Yat‐sen Memorial Hospital Sun Yat‐sen University Guangzhou People's Republic of China; ^3^ Guangdong Province Key Laboratory of Brain Function and Disease Zhongshan School of Medicine Sun Yat‐sen University Guangzhou People's Republic of China

Dear Editor,

Radiation‐induced brain necrosis (RN) may occur in 3%–24% of patients who receive radiotherapy for head and neck tumors.^1^ Corticosteroids have long been viewed as the first‐line treatment for RN.^2^ Bevacizumab has been proved to be superior to the classic corticosteroids treatment by many studies and is being used increasingly, but with potential toxicity.^3^, ^4^, ^5^ Neutrophil/lymphocyte ratio (NLR), platelet/lymphocyte ratio (PLR), monocyte/lymphocyte ratio (MLR) and mean platelet volume (MPV) are widely used systemic inflammation indicators and have been well studied in their efficacy in predicting prognosis in multiple researches.^6^ This study aimed to explore the changes in these four biomarkers during bevacizumab or corticosteroids treatment and their potential predictive value of treatment response.

Our study included 110 patients that were diagnosed with RN after radiation therapy for nasopharyngeal cancer and treated with bevacizumab, and additional 169 RN patients received corticosteroids treatment. Table 1 and Table S1 present the basic patient information. All detailed methods were provided in the Supporting Materials. We first looked into NLR, MLR, PLR and MPV changes during bevacizumab treatment (Figure 1). NLR level decreased during bevacizumab treatment in all patients overall (Figure 1A). In the effective group, a similar trend was observed while statistics difference was only seen between baseline and treatment 3 (Figure 1B). On the other hand, NLR level decreased between each treatment with baseline in the ineffective group (Figure 1C). Focusing on the individual effect of bevacizumab treatment, we found that NLR decreased after three courses of treatment compared with that at baseline in all patients, the effective and ineffective groups (Figure 1D‐F). Interestingly, MLR decreased after three courses of bevacizumab treatment in all patients and the ineffective groups, but not in the effective group (Figure 1G‐L). In addition, no significant change was observed in PLR or MPV after treatment 3 in all patients, the effective or ineffective group (Figure S1).

**TABLE 1:** Baseline clinical characteristics of the patients

Variables	Bevacizumab group (*n* = 110)	Corticosteroids group (*n* = 169)
**Age** (years)	48 (42–56)	49 (44–55)
**Sex**		
Male	81 (73.6)	137 (81.1)
Female	29 (26.4)	32 (18.9)
**WBC** (10^∧^9/L)	5.5 (4.4–6.9)	6.6 (5.2–8.7)
**Lymphocyte** (10^∧^9/L)	1.2 (1.0–1.5)	1.1 (.8–1.4)
**Neutrophil** (10^∧^9/L)	3.6 (2.5–4.9)	5.1 (3.5–7.2)
**Monocytes** (10^∧^9/L)	.4 (.3–.5)	.3 (.1–.4)
**Platelet** (10^∧^9/L)	215.5 (184.2–258.0)	226.0 (191.0–271.0)
**Baseline NLR**	2.931 (2.123–4.176)	5.052 (2.662–7.638)
**NLR2** ^†^	2.473 (1.898–3.564)	3.970 (2.480–6.333)
**NLR3** ^†^	2.426 (1.728–3.565)	‐
**NLR4** ^†^	2.423 (1.680–3.597)	‐
**Baseline MLR**	.304 (.235–.421)	.226 (.088–.318)
**MLR2** ^†^	.301 (.223–.404)	.265 (.157–.346)
**MLR3** ^†^	.269 (.194–.362)	‐
**MLR4** ^†^	.259 (.184–.362)	‐
**Baseline PLR**	184.084 (139.898–238.746)	203.571 (159.375–292.208)
**PLR2** ^†^	172.036 (135.305–244.118)	185.271 (141.304–266.667)
**PLR3** ^†^	171.111 (129.631–218.473)	‐
**PLR4** ^†^	162.270 (130.957–230.468)	‐
**Baseline MPV** (μm^3^)	9.900 (9.225–10.500)	10.200 (9.700–10.700)
**MPV2** ^†^ (μm^3^)	9.800 (9.200–10.300)	10.200 (9.600–10.800)
**MPV3** ^†^ (μm^3^)	9.900 (9.300–10.300)	‐
**MPV4** ^†^ (μm^3^)	9.900 (9.400–10.400)	‐
**LDH** (U/L)	166.5 (149.0–199.0)	206.0 (182.0–239.0)
**hs‐CRP** (mg/L)	2.0 (.7–6.1)	3.1 (1.2–8.5)
**LENT/SOMA**		
Grade 1	21 (19.1)	78 (46.1)
Grade 2	31 (28.2)	27 (16.0)
Grade 3	41 (37.3)	41 (24.3)
Grade 4	17 (15.4)	23 (13.6)
**MoCA**	24.0 (22.0–27.0)	24.0 (22.0–26.0)
**T stage**		
1	2 (1.8)	40 (23.7)
2	14 (12.7)	18 (10.7)
3	58 (52.8)	67 (39.6)
4	36 (32.7)	44 (26.0)
**N stage**		
0	19 (17.3)	63 (37.3)
1	54 (49.1)	69 (40.8)
2	30 (27.3)	36 (21.3)
3	7 (6.3)	1 (.6)
TNM Stage^*^		
I	1 (.9)	0 (0)
II	7 (6.4)	9 (5.3)
III	63 (57.3)	74 (43.8)
IVA	39 (35.4)	86 (50.9)
**IRB** (months)	46.2 (29.2–62.8)	52.2 (37.6–54.0)
**IBT** (months)	3.7 (.8–12.7)	6.9 (.1–25.9)
**D_max_ to the brain** (Gy)	70.0 (68.0–70.0)	70.0 (70.0–72.0)
**Total radiation dose to the neck** (Gy)	60.0 (56.0–66.0)	60.0 (58.0–64.0)
**Radiation approach**		
Conventional radiotherapy	50 (45.5)	149 (88.2)
IMRT	60 (54.5)	20 (11.8)
**Lesion volume** (cm^3^)	36.0 (14.8–80.6)	16.9 (4.6–50.6)
**Decrease in lesion volume** (%)	62.6 (28.8–84.5)	2.2 (−36.9–29.6)
**Treatment response**		
Ineffective	26 (23.6)	118 (69.8)
Effective	84 (76.4)	51 (30.2)

*Note*: The data are shown as the number (percentage) or median (interquartile range).

Abbreviations: D_max_, maximum radiation dose; hs‐CRP, high‐sensitivity C‐reactive protein; IBT, interval between diagnosis of brain necrosis and treatment with bevacizumab; IMRT, intensity‐modulated radiation therapy; IRB, interval between radiotherapy and diagnosis of brain necrosis; LDH, lactate dehydrogenase; LENT/SOMA, late effects of normal tissue subjective, objective, management; MLR, monocyte to lymphocyte ratio; MoCA, Montreal cognitive assessment; MPV, mean platelet volume; NLR, neutrophil to lymphocyte ratio; PLR, platelet to lymphocyte ratio; WBC, white blood cell count.

^†^ In bevacizumab cohort, bevacizumab was administered once every 2 weeks for four courses, that is, bevacizumab was delivered at week 0, week 2, week 4 and week 6 (as named treatment 1, 2, 3 and 4, respectively). NLR2, NLR3 and NLR4 refer to NLR levels before bevacizumab treatment 2, 3 and 4, respectively; the same is true for MLR2 to 4, PLR2 to 4 and MPV2 to 4. In corticosteroids cohort, NLR2, MLR2, PLR2 and MPV2 refer to NLR, MLR, PLR and MPV levels after corticosteroids treatment.

*TNM (Tumour‐Node‐Metastasis) stage was classified according to the AJCC 8th TNM staging system.

**FIGURE 1:**
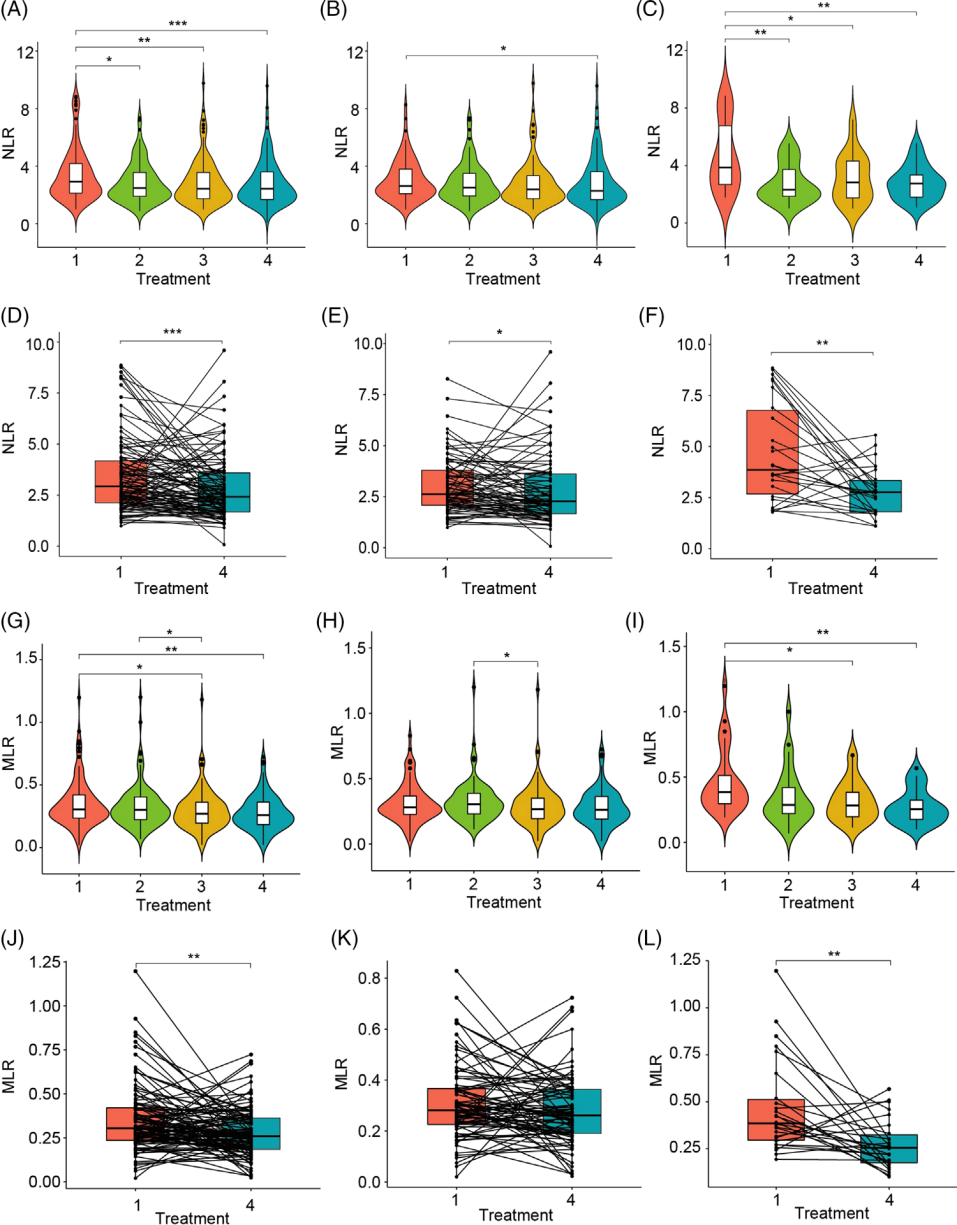
**Neutrophil to lymphocyte ratio (NLR) change during bevacizumab treatment**. (A‐C) Violin plots to show NLR change in different course of bevacizumab treatment in all patients (A), the effective group (B) and the ineffective group (C), respectively. (D‐F) Paired box plots to show NLR change after three courses of treatment in every single individual in all patients (D), the effective group (E) and the ineffective group (F), respectively. (G‐I) Violin plots to show monocyte to lymphocyte ratio (MLR) change in different course of bevacizumab treatment in all patients (G), the effective group (H) and the ineffective group (I), respectively. (J‐L) Paired box plots to show MLR change after three courses of treatment in every single individual in all patients (J), the effective group (K) and the ineffective group (L), respectively. In bevacizumab cohort, bevacizumab was administered once every 2 weeks for four courses, that is, bevacizumab was delivered at week 0, week 2, week 4 and week 6 (as named treatment 1, 2, 3, and 4, respectively). ^*^ *p* < .05. ^**^ *p* < .01. ^***^ *p* < .001

We further investigated the efficacy of baseline NLR and MLR in stratifying patients by treatment response (Figure 2). In the effective group, both baseline NLR and MLR were lower than those in the ineffective group separately (Figure 2A,B). Moreover, the area under the curve (AUC) analysis showed favourable discrimination of baseline NLR (AUC .699, 95% CI .574–.825, Figure 2C) and baseline MLR (AUC .708, 95% CI .597–.820, Figure 2D).

**FIGURE 2:**
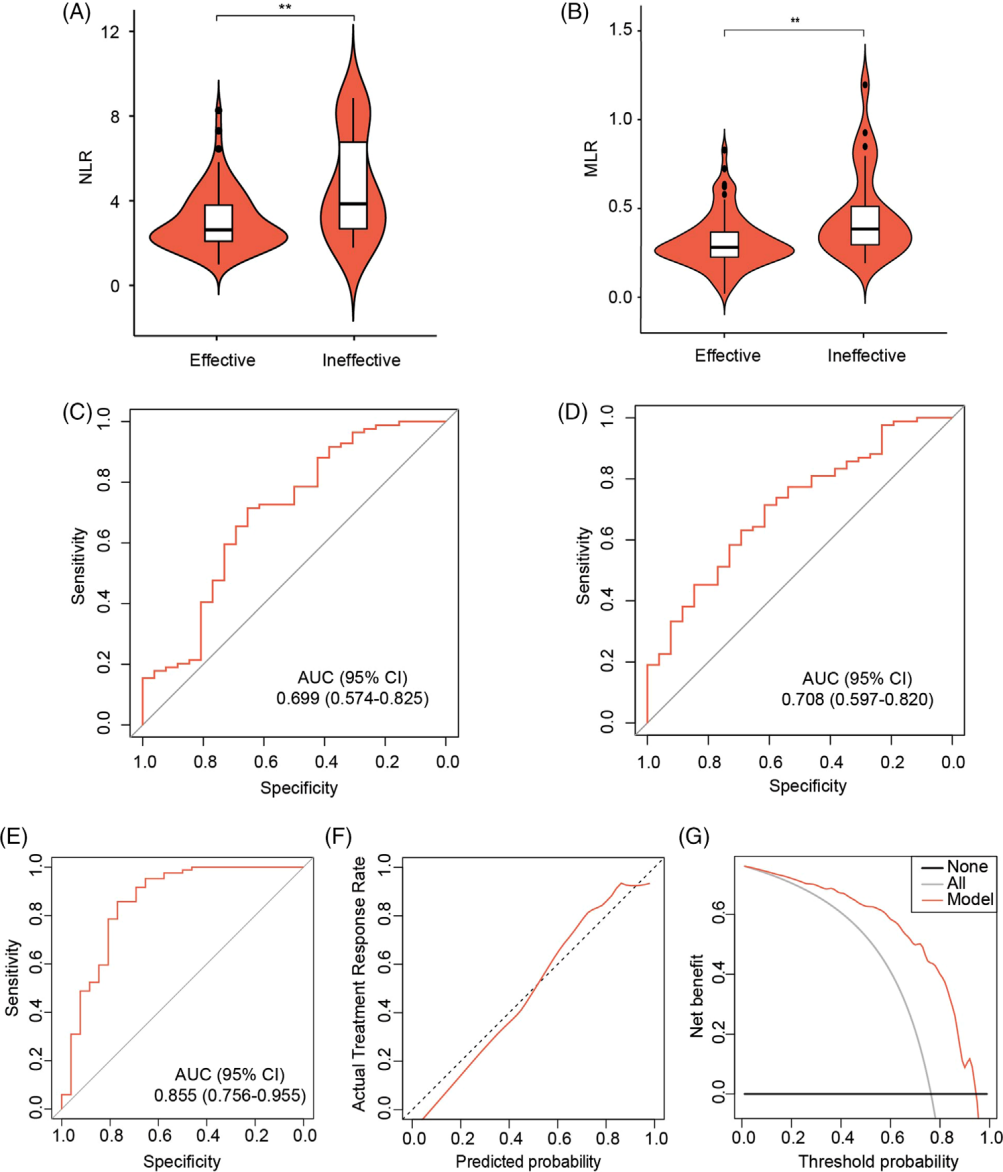
**Predictive efficacy of baseline neutrophil to lymphocyte ratio (NLR) and monocyte to lymphocyte ratio (MLR), and performance evaluation of the prediction model in patients treated with bevacizumab**. (A and B) Comparison of baseline NLR (A) and MLR (B) in the effective and the ineffective groups. (C and D) Receiver operator characteristic (ROC) curves of baseline NLR (C) and MLR (D) for prediction of bevacizumab treatment response. (E) ROC curve of the prediction model. (F) Calibration curve of the prediction model. The calibration curves depict the calibration of the model in terms of agreement between the predicted probability of a treatment response and the observed treatment outcome. The 45‐degree dotted line represents a perfect prediction by an ideal model, and the solid line represents the performance of the model, of which a closer fit to the diagonal dotted line represents a better prediction. (G) Decision curve analysis for the prediction model. The grey line represents the assumption that all patients had a favourable response to treatment. The black line represents the assumption that no patient had a favourable response. The red line represents the model. The decision curve reveals that if the threshold probability is <94%, using the proposed model to detect the treatment response is more advantageous than either the treat‐all regimen or the treat‐none regimen. ^**^ *p* < .01

Furthermore, we constructed a prediction model for treatment response to bevacizumab using multivariable logistic regression. As a result, baseline NLR, the interval between diagnosis of brain necrosis and treatment with bevacizumab (IBT), and the interval between radiotherapy (IRB) and diagnosis of brain necrosis were identified as independent predictors (Table 2 and Table S2). The calculation formula for the response score was shown below: 5.520–.535 × baseline NLR – .025 × IRB – .078 × IBT. The predicted probability of effective treatment response was calculated using 1/(1 + exp [−response score]).

**TABLE 2:** Univariable and multivariable logistic regression of potential predictors of the response to bevacizumab in patients with brain necrosis

Variables	Univariable logistic regression		Multivariable logistic regression	
	OR (95% CI)	*p*‐value	OR (95% CI)	*p*‐value
**Age** (years)	.992 (.947–1.039)	.731	‐	‐
**Sex** (Male vs. Female)	.468 (.183–1.221)	.113	‐	‐
**Baseline NLR**	.632 (.484–.802)	.001*	.586 (.415–.788)	<.001*
**Baseline MLR**	.017 (.001–.178)	.001*	‐	‐
**Baseline PLR**	.996 (.991–1.000)	.047*	‐	‐
**Baseline MPV**	.877 (.133–1042.907)	.555	‐	‐
**WBC** (10^∧^9/L)	.719 (.549–.927)	.012*	‐	‐
**LDH** (U/L)	.984 (.972–.995)	.007*	‐	‐
**hs‐CRP** (mg/L)	.996 (.968–1.032)	.805	‐	‐
**LENT/SOMA**				
Grade 1	Reference	‐	‐	‐
Grade 2	.676 (.159–2.530)	.572	‐	‐
Grade 3	.971 (.232–3.564)	.965	‐	‐
Grade 4	.431 (.091–1.853)	.264	‐	‐
**MoCA**	1.081 (.973–1.202)	.142	‐	‐
**IRB** (months)	.977 (.963–.989)	<.001*	.976 (.957–.992)	.007*
**IBT** (months)	.935 (.898–.966)	<.001*	.925 (.876–.968)	.002*
**D_max_ to the brain** (Gy)	1.017 (.914–1.119)	.742	‐	‐
**Total radiation dose to the neck** (Gy)	1.003 (.940–1.058)	.907	‐	‐
**Radiation approach** (Conventional radiotherapy vs. IMRT)	1.038 (.424–2.512)	.935	‐	‐

Abbreviations: CI, confidence interval; D_max_, maximum radiation dose; hs‐CRP, high‐sensitivity C‐reactive protein; IBT, interval between diagnosis of brain necrosis and treatment with bevacizumab; IMRT, intensity‐modulated radiation therapy; IRB, interval between radiotherapy and diagnosis of brain necrosis; LDH, lactate dehydrogenase; LENT/SOMA, late effects of normal tissue subjective, objective, management; MLR, monocyte to lymphocyte ratio; MoCA, Montreal cognitive assessment; MPV, mean platelet volume; NLR, neutrophil to lymphocyte ratio; OR, odds ratio; PLR, platelet to lymphocyte ratio; WBC, white blood cell count.

* *p* < .05.

Subsequently, we evaluated the performance of the prediction model. The optimal cut‐off values of the response score, baseline NLR, IRB and IBT were determined as .940, 3.571, 49.4 and 27.0. The model showed satisfactory discrimination (AUC .855, 95% CI .756–.955, Figure 2E). In addition, the calibration curve demonstrated good calibration of the model (Figure 2F). The Hosmer–Lemeshow test indicated no deviation from the perfect match with a *p* value of .317. Moreover, the decision curve analysis demonstrated that the model is clinically useful (Figure 2G). This model could inform a clinician how big the possibility is that a certain patient would respond to bevacizumab treatment and avoid adverse effects brought by bevacizumab on patients that would not respond well.

In the meantime, we also studied how these biomarkers changed after corticosteroids treatment. We found that NLR decreased and MLR increased after treatment, while PLR and MPV did not change significantly in all patients overall; subgroup analysis showed that MLR increased and PLR decreased in the ineffective group (Figure S2). Moreover, baseline NLR, MLR, PLR or MPV displayed no significant difference between the effective and ineffective groups (Figure S3) and were not predictors for corticosteroids treatment response (Table S3).

We hypothesized that the association between NLR and bevacizumab treatment may be relevant to the pathogenesis of RN. Although the pathogenesis of RN largely remains unknown, endothelial cell dysfunction has been proposed as the primary cause.^7^, ^8^ In brief, radiation‐induced endothelial damage leads to blood‐brain barrier (BBB) destruction, and hypoxia and generation of hypoxia‐inducible factor‐1α in local tissue, which strongly mediates the upregulation of vascular endothelial growth factor (VEGF).^7^, ^8^ Additionally, the breakdown of BBB would lead to peripheral immune cells infiltrating into the brain and leak out of antigen from central nervous system to the peripheral blood, both could activate the immune system. Lymphocytes have been found to infiltrate into the central nervous system and are partly responsible for neuronal damage and clinical symptoms.^9^, ^10^ Bevacizumab treatment would lead to BBB normalization which may decrease the leak out of antigen and infiltration of immune cells including both neutrophils and lymphocytes.^8^ In this study, patients with lower baseline NLR tend to have positive responses to bevacizumab treatment which can reduce VEGF. This may indicate a larger role of VEGF elevation and BBB breakdown in the pathophysiology in these patients. Apart from NLR, other independent predictors (i.e., IRB and IBT) are negatively correlated with bevacizumab treatment response, which is interpretable since the shorter duration after pathological changes would naturally indicate less irreversible damage of the brain thus leading to better treatment outcomes.

In conclusion, we confirmed the predictive role of NLR in treatment response to bevacizumab in RN patients and constructed a reliable prediction model by integrating NLR, IRB and IBT. Additional external and prospective studies are needed to validate the prediction model as well as to investigate the role of immunity and BBB in the pathogenies of RN, which could provide potential intervention targets in the future.

## CONFLICT OF INTEREST

The authors declare no potential conflict of interest.

## Supporting information



Supporting Information
