# Supplementary material for: Neutrophil to lymphocyte ratio as a predictor for treatment of radiation‐induced brain necrosis with bevacizumab in nasopharyngeal carcinoma patients
Source: Clin Transl Med. 2022 Jan 24;12(1):e583. doi: 10.1002/ctm2.583 (PMC8787024; doi:10.1002/ctm2.583)

# Supplementary Materials

**Supplementary Methods ........................................................................................2**

Patients ................................................................................................2

Inclusion and exclusion criteria ...........................................................................2

Diagnosis of radiation-induced brain necrosis......................................................2

Treatment and evaluations ....................................................................................3

Definition of effective treatment response ............................................................3

Statistical analysis .................................................................................................4

**Supplementary Tables ..........................................................................................6**

Table S1. Baseline laboratory test results of the patients before treatment with bevacizumab or corticosteroids.............................................................................6

Table S2. Univariable logistic regression of potential predictors of the response to bevacizumab. .........................................................................................................7

Table S3. Univariable logistic regression of NLR, MLR, PLR and MPV in corticosteroid treatment. ........................................................................................8

**Supplementary Figures .........................................................................................9**

Figure S1. PLR and MPV changes during bevacizumab treatment in subgroups. .9

Figure S2. NLR, MLR, PLR and MPV changes after corticosteroids treatment.. 11

Figure S3. Comparison of baseline NLR, MLR, PLR and MPV in corticosteroids treatment subgroups. ...........................................................................................12

## Supplementary Methods

### Patients

This retrospective study was approved by ethics committee in Sun Yat-sen memorial hospital. Informed consent was signed in all participating patients before recruitment. In our hospital, patients who were diagnosed with radiation-induced brain necrosis (RN) after radiotherapy for nasopharyngeal carcinoma (NPC) and received bevacizumab treatment between July 2012 and November 2019, and additional patients received corticosteroids treatment between January 2005 and December 2016 were enrolled[1]. All the patients were staged according to the 8th edition of UICC/AJCC TNM staging system[2].

### Inclusion and exclusion criteria

In the bevacizumab cohort, the inclusion criteria were as follows: (1) histologically confirmed nasopharyngeal carcinoma patients subjected to radiotherapy; (2) radiographic evidence supporting a diagnosis of RN; (3) fulfilled four courses of bevacizumab treatment; (4) not previously administered corticosteroids; (5) lacked evidence regarding increased intracranial pressure suggestive of brain hernia requiring surgical intervention; and (6) MRI was performed before and after treatment. The exclusion criteria were as follows: (1) incomplete clinical data such as missing blood routine at baseline or during visit or untraceable record of radiotherapy schemes; (2) with tumor recurrence or metastases; and (3) with infectious condition in lung, liver or urinary tract or other systems.

In the corticosteroids cohort, the inclusion criteria were as follows: (1) histologically confirmed nasopharyngeal carcinoma patients subjected to radiotherapy; (2) radiographic evidence supporting a diagnosis of RN; (3) treated using intravenous methylprednisolone (high-dose or low-dose); (4) not previously administered bevacizumab; (5) lacked evidence regarding increased intracranial pressure suggestive of brain hernia requiring surgical intervention; and (6) MRI was performed before and after treatment. The exclusion criteria were as follows: (1) incomplete clinical data such as missing blood routine at baseline or during visit or untraceable record of radiotherapy schemes; (2) with tumor recurrence or metastases; and (3) with infectious condition in lung, liver or urinary tract or other systems.

### Diagnosis of radiation-induced brain necrosis

The diagnosis of radiation necrosis is mainly based on opinions from both neurologists and radiologists as described in previous studies[3, 4]. Patients with

history of radiotherapy for cancer, must showed neurologic symptoms or signs appropriate to the location of the brain necrosis which were graded with the Late Effects of Normal Tissue/Subjective, Objective, Management, Analytic (LENT/SOMA) scale. In addition, the radiographic change must display a high-intensity lesion on fluid-attenuated inversion recovery (FLAIR) imaging and a lesion of enhancement on post-gadolinium imaging, especially “soap bubble” or “Swiss cheese” enhancement; when necessary, the diagnosis was confirmed by positron emission tomography/computed tomography (PET/ CT) imaging or biopsy [3, 4].

**Treatment and evaluations**

In the bevacizumab cohort, bevacizumab (Avastin, Genentech, South San Francisco, CA, USA) was administered at 5 mg/kg intravenously once every 2 weeks for 4 courses, i.e., bevacizumab was delivered at week 0, week 2, week 4 and week 6 (as named treatment 1, 2, 3 and 4, respectively). Before each treatment, laboratory tests were ordered including complete blood cell counts with differential count, serum chemistry test and coagulation routine; neurologic signs and symptoms were assessed by the LENT/SOMA scales; cognitive function was evaluated using the Montreal Cognitive Assessment (MoCA, Chinese version). The LENT/SOMA scale contains four domains. Each domain scores from 1 to 4, and a 0 score indicates there are no toxicities. We defined the final grade of the LENT/SOMA scale as the maximum score of the 4 components. A reduction ≥1 in the grade of the LENT/SOMA scale was defined as an improvement [5-7]. The MoCA contains seven cognitive domains. Each domain has a maximum score of 2–6, and the final MoCA score is defined as the total score across all seven domains [8]. The volumes of the brain lesions were detected by FLAIR imaging within 3 days before the initiation of treatment and 2 weeks after completion of treatment.

In the corticosteroids cohort, patients were treated with low-dose or high-dose methylprednisolone. Previously we found that there were no significant differences in the treatment response based on lesion volume on MRI, or changes in clinical symptoms and cognitive function between low-dose and high-dose regimens[1]. Low dose methylprednisolone was administered as an intravenous infusion of 1 mg/kg/day for 5 consecutive days, 40 mg for 5 days, then oral prednisone 30 mg per day, gradually tapering by 5 mg/week to a maintenance dose of 10 mg daily for 3 months. High-dose methylprednisolone was administered as an intravenous infusion of 500 mg for 3 consecutive days, 80 mg for 4 days, 40 mg for 4 days, then oral prednisone 30 mg per day, gradually tapering by 5 mg/week to a maintenance dose of 10 mg daily for 3 months. Laboratory tests, assessments and MRI examination were conducted before and after treatment (as named treatment 1 and 2, respectively).

**Definition of effective treatment response**

The effective treatment response was defined as (A) an improvement in neurologic manifestations (a reduction ≥1 in the grade of the LENT/SOMA scale was defined as an improvement) [4, 6, 7]; or (B) stable neurologic manifestations and a reduction in lesion volume on FLAIR images by ≥25% [4].

**Statistical analysis**

Data were presented using descriptive statistics (relative frequencies for categorical variables, median and interquartile range [IQR] for continuous variables). Paired Wilcoxon signed-rank test was used to compared baseline NLR, MLR, PLR or MPV with that in each visit in all patients, the effective and the ineffective groups. Mann-Whitney *U* tests were performed to assess the potential association of baseline NLR, MLR, PLR or MPV with the treatment response. The discrimination performance of potential predictors was evaluated by the area under the receiver operator characteristic (ROC) curve (AUC).

Univariable logistic regression was performed between each candidate variable and treatment outcome. Those variables with $P < 0.05$ in univariable logistic regression were then included to multivariable logistic regression using likelihood ratio test with backward step-down selection. The collinearity diagnostics of the multivariable logistic regression was estimated by a variance inflation factor (VIF). In this study, the VIF values ranged from 1.096 to 3.895, indicating that there was no collinearity in the collinearity diagnosis.

A response score was calculated based on the results of multivariable logistic regression by a liner combination of predictors weighted by their corresponding coefficients, which can be used as a prediction model to reflect the probability of treatment response for each patient[9]. The discrimination performance of the prediction model was measured by an AUC[10]. The optimal cut-off values for the response score and each predictor were determined based on maximum Youden index. A calibration curve was plotted to evaluate the calibration of the prediction model, along with the Hosmer-Lemeshow test to measure the goodness-of-fit [11]. Decision curve analysis (DCA) was conducted to estimate the clinical usefulness of the model by quantifying the net benefits at different threshold probabilities[12].

All statistical tests were performed using R statistical software (version 3.5.2; R Foundation for Statistical Computing). The "*pROC*" package was applied to plot ROC curves. The VIF values were evaluated using the "*car*" package. The "*rms*" package was used to plot calibration curve. The Hosmer-Lemeshow test was performed using the "*vcdExtra*" package. DCA was plotted using the "*dca.R*". A two-sided $P < 0.05$ was considered statistically significant.

**References**

1. Zhuo X, Huang X, Yan M et al. Comparison between high-dose and low-dose intravenous methylprednisolone therapy in patients with brain necrosis after radiotherapy for nasopharyngeal carcinoma. Radiother Oncol 2019; 137: 16-23.
2. Amin MB, Edge SB, Greene FL et al. AJCC cancer staging manual, 8th edn. New York, NY: Springer, 2017.
3. Boothe D, Young R, Yamada Y et al. Bevacizumab as a treatment for radiation necrosis of brain metastases post stereotactic radiosurgery. Neuro-Oncology 2013; 15: 1257-1263.
4. Levin VA, Bidaut L, Hou P et al. Randomized double-blind placebo-controlled trial of bevacizumab therapy for radiation necrosis of the central nervous system. Int J Radiat Oncol Biol Phys 2011; 79: 1487-1495.
5. LENT SOMA scales for all anatomic sites. Int J Radiat Oncol Biol Phys 1995; 31: 1049-1091.
6. Xu Y, Rong X, Hu W et al. Bevacizumab Monotherapy Reduces Radiation-induced Brain Necrosis in Nasopharyngeal Carcinoma Patients: A Randomized Controlled Trial. Int J Radiat Oncol Biol Phys 2018; 101: 1087-1095.
7. Nutting CM, Morden JP, Harrington KJ et al. Parotid-sparing intensity modulated versus conventional radiotherapy in head and neck cancer (PARSPORT): a phase 3 multicentre randomised controlled trial. The Lancet Oncology 2011; 12: 127-136.
8. Nasreddine ZS, Phillips NA, Bedirian V et al. The Montreal Cognitive Assessment, MoCA: a brief screening tool for mild cognitive impairment. J Am Geriatr Soc 2005; 53: 695-699.
9. Wu S, Zheng J, Li Y et al. A Radiomics Nomogram for the Preoperative Prediction of Lymph Node Metastasis in Bladder Cancer. Clin Cancer Res 2017; 23: 6904-6911.
10. Han K, Song K, Choi BW. How to Develop, Validate, and Compare Clinical Prediction Models Involving Radiological Parameters: Study Design and Statistical Methods. Korean J Radiol 2016; 17: 339-350.
11. Kramer AA, Zimmerman JE. Assessing the calibration of mortality benchmarks in critical care: The Hosmer-Lemeshow test revisited. Crit Care Med 2007; 35: 2052-2056.
12. Vickers AJ, Elkin EB. Decision curve analysis: a novel method for evaluating prediction models. Med Decis Making 2006; 26: 565-574.

## Supplementary Tables

### Table S1. Baseline laboratory test results of the patients before treatment with bevacizumab or corticosteroids.

| Variables | Bevacizumab group (n = 110) | Corticosteroids group (n = 169) |
|---|---|---|
| **RBC** (10^12/L) | 4.5 (4.1-4.8) | 4.5 (4.2-5.0) |
| **HGB** (g/L) | 132.5 (121.2-139.0) | 135.0 (125.0-145.0) |
| **AST** (U/L) | 20.0 (16.3-25.0) | 20.0 (17.0-25.0) |
| **ALT** (U/L) | 18.0 (13.0-24.0) | 19.0 (14.0-27.0) |
| **TB** (μmol/L) | 10.9 (7.8-12.3) | 9.8 (8.0-11.6) |
| **Na** (mmol/L) | 140.3 (139.1-141.5) | 140.0 (138.6-141.5) |
| **K** (mmol/L) | 4.0 (3.8-4.2) | 4.1 (3.9-4.3) |
| **Urea** (mmol/L) | 4.2 (3.6-5.2) | 4.5 (3.8-5.2) |
| **CREA** (μmol/L) | 84.0 (71.0-92.8) | 93.0 (83.0-104.0) |
| **GLU** (mmol/L) | 4.7 (4.2-5.0) | 5.6 (4.6-7.1) |
| **TC** (mmol/L) | 5.0 (4.4-5.5) | 5.4 (4.9-6.1) |
| **TG** (mmol/L) | 1.1 (0.8-1.5) | 1.0 (0.7-1.4) |
| **HDL** (mmol/L) | 1.2 (1.1-1.5) | 1.3 (1.2-1.6) |
| **LDL** (mmol/L) | 3.1 (2.7-3.7) | 3.4 (3.0-3.9) |
| **apoA1** (g/L) | 1.2 (1.0-1.3) | 1.1 (1.0-1.3) |
| **apoB** (g/L) | 0.9 (0.8-1.0) | 0.9 (0.8-1.0) |
| **ALB** (g/L) | 39.3 (37.6-41.3) | 41.2 (38.8-43.8) |
| **GLB** (g/L) | 25.8 (23.5-27.9) | 24.4 (22.4-27.4) |
| **ALP** (U/L) | 81.0 (55.3-107.0) | 87.0 (59.0-126.0) |
| **PT** (s) | 11.6 (11.0-12.1) | 11.3 (10.8-11.8) |
| **APTT** (s) | 27.3 (24.2-29.9) | 26.0 (23.7-30.0) |

The data are shown as the median (interquartile range).

**Abbreviations**: ALB, albumin; ALP, alkaline phosphatase; ALT, alanine aminotransferase; apoA1, apolipoprotein A1; apoB, apolipoprotein B; APTT, activated partial thromboplastin time; AST, aspartate aminotransferase; CREA, Creatinine; GLB, globulin; GLU, glucose; HDL, high density lipoprotein; HGB, hemoglobin; K, potassium; LDL, low density lipoprotein; Na, sodium; PT, prothrombin time; RBC, red blood cell count; TB, total bilirubin; TC, Cholesterol; TG, triglycerides; Urea, blood urea nitrogen.

**Table S2. Univariable logistic regression of potential predictors of the response to bevacizumab.**

| Variables | Univariable logistic regression | |
|---|---|---|
| | OR (95% CI) | *P*-value |
| **RBC** (10^12/L) | 1.091 (0.505-2.378) | 0.823 |
| **HGB** (g/L) | 0.990 (0.959-1.020) | 0.517 |
| **AST** (U/L) | 1.011 (0.956-1.081) | 0.729 |
| **ALT** (U/L) | 0.996 (0.971-1.028) | 0.796 |
| **TB** ( μ mol/L) | 0.986 (0.914-1.078) | 0.726 |
| **Na** (mmol/L) | 1.055 (0.883-1.256) | 0.544 |
| **K** (mmol/L) | 1.700 (0.834-6.637) | 0.428 |
| **Urea** (mmol/L) | 1.182 (0.823-1.742) | 0.378 |
| **CREA** (μmol/L) | 1.021 (0.991-1.053) | 0.191 |
| **GLU** (mmol/L) | 0.975 (0.588-1.695) | 0.924 |
| **TC** (mmol/L) | 1.068 (0.698-1.651) | 0.763 |
| **TG** (mmol/L) | 1.146 (0.677-2.260) | 0.648 |
| **HDL** (mmol/L) | 0.487 (0.126-1.918) | 0.294 |
| **LDL** (mmol/L) | 1.080 (0.646-1.832) | 0.770 |
| **apoA1** (g/L) | 0.982 (0.346-4.142) | 0.975 |
| **apoB** (g/L) | 0.737 (0.092-5.756) | 0.770 |
| **ALB** (g/L) | 1.138 (1.007-1.305) | 0.051 |
| **GLB** (g/L) | 1.045 (0.941-1.165) | 0.413 |
| **ALP** (U/L) | 1.002 (0.995-1.012) | 0.582 |
| **PT** (s) | 0.852 (0.512-1.225) | 0.484 |
| **APTT** (s) | 1.033 (0.931-1.154) | 0.544 |

**Abbreviations**: ALB, albumin; ALP, alkaline phosphatase; ALT, alanine aminotransferase; apoA1, apolipoprotein A1; apoB, apolipoprotein B; APTT, activated partial thromboplastin time; AST, aspartate aminotransferase; CI, confidence interval; CREA, Creatinine; GLB, globulin; GLU, glucose; HDL, high density lipoprotein; HGB, hemoglobin; K, potassium; LDL, low density lipoprotein; Na, sodium; OR, odds ratio; PT, prothrombin time; RBC, red blood cell count; TB, total bilirubin; TC, Cholesterol; TG, triglycerides; Urea, blood urea nitrogen.

**Table S3. Univariable logistic regression of NLR, MLR, PLR and MPV in corticosteroid treatment.**

| Variables | Univariable logistic regression | |
|---|---|---|
| | OR (95% CI) | *P*-value |
| **NLR** | 1.015 (0.930-1.105) | 0.725 |
| **MLR** | 0.461 (0.059-3.177) | 0.444 |
| **PLR** | 1.001 (0.998-1.004) | 0.576 |
| **MPV** | 1.098 (0.754-1.591) | 0.620 |

**Abbreviations**: CI, confidence interval; MLR, monocyte to lymphocyte ratio; MPV, mean platelet volume；NLR, neutrophil to lymphocyte ratio; OR, odds ratio; PLR, platelet to lymphocyte ratio.

## Supplementary Figures

### Figure S1. PLR and MPV changes during bevacizumab treatment in subgroups.

(A-C) Violin plots to show PLR change after bevacizumab treatment in all patients (A), the effective (B) and the ineffective (C) groups.

(D-F) Paired box plots to show PLR change after three courses of treatment in every single individual in all patients (D), the effective (E) and the ineffective (F) groups.

(G-I) Violin plots to show MPV changes after bevacizumab treatment in all patients (G), the effective (H) and the ineffective (I) groups.

(J-L) Paired box plots to show MPV change after three courses of treatment in every single individual in all patients (J), the effective (K) and the ineffective (L) groups.

In bevacizumab cohort, bevacizumab was administered once every 2 weeks for 4 courses, i.e., bevacizumab was delivered at week 0, week 2, week 4 and week 6 (as named treatment 1, 2, 3 and 4, respectively).

$^{*}P < 0.05$. $^{**}P < 0.01$.

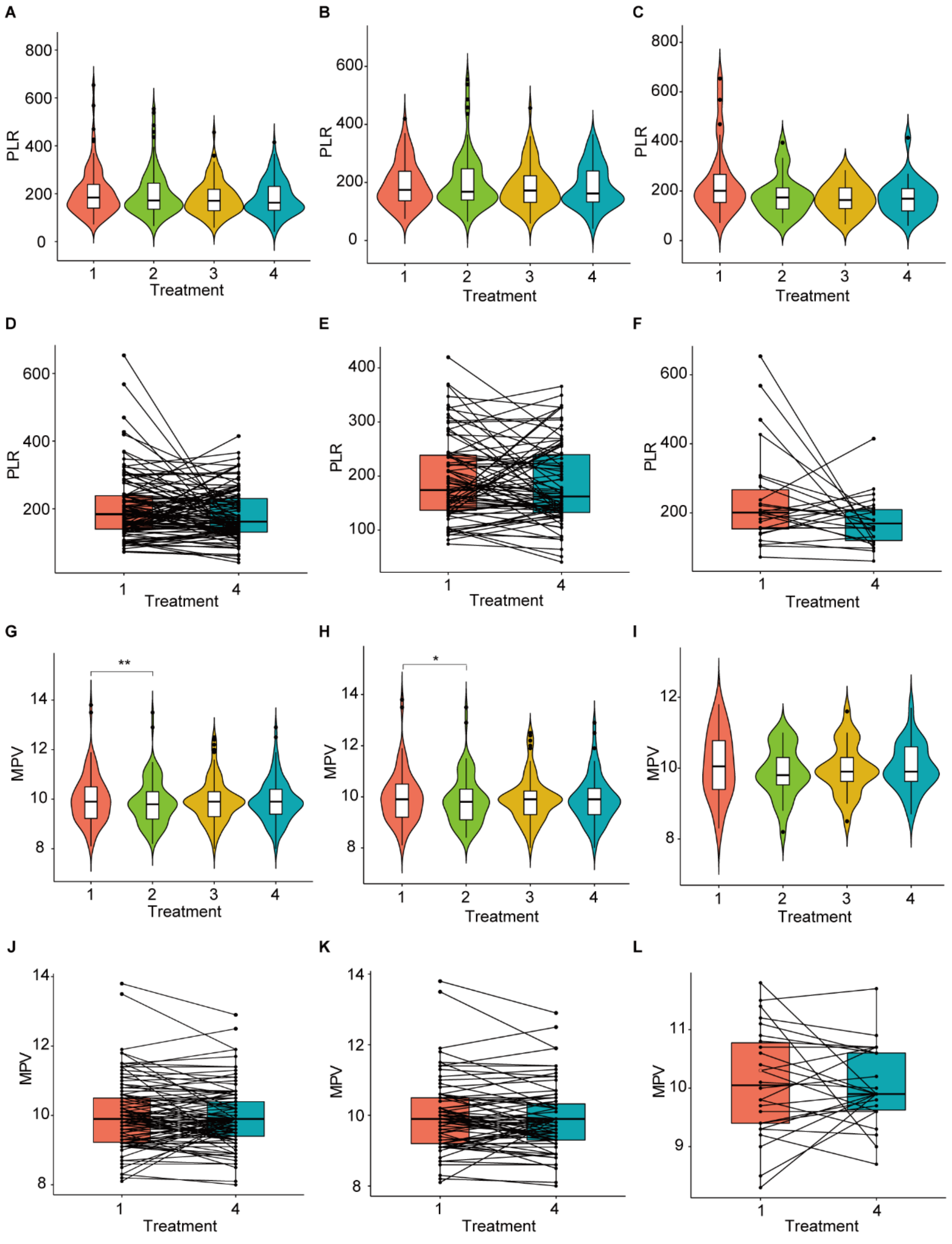
A
B
C
D
E
F
G
H
I
J
K
L
PLR
MPV
Treatment
**
*

**Figure S2. NLR, MLR, PLR and MPV changes after corticosteroids treatment.**

Violin plots to show NLR (A-C), MLR (D-F), PLR (G-I) and MPV (J-L) changes after corticosteroids treatment in all patients (A, D, G and J), the effective group (B, E, H and K) and the ineffective group (C, F, I and L), respectively.

$^{*}P < 0.05$.

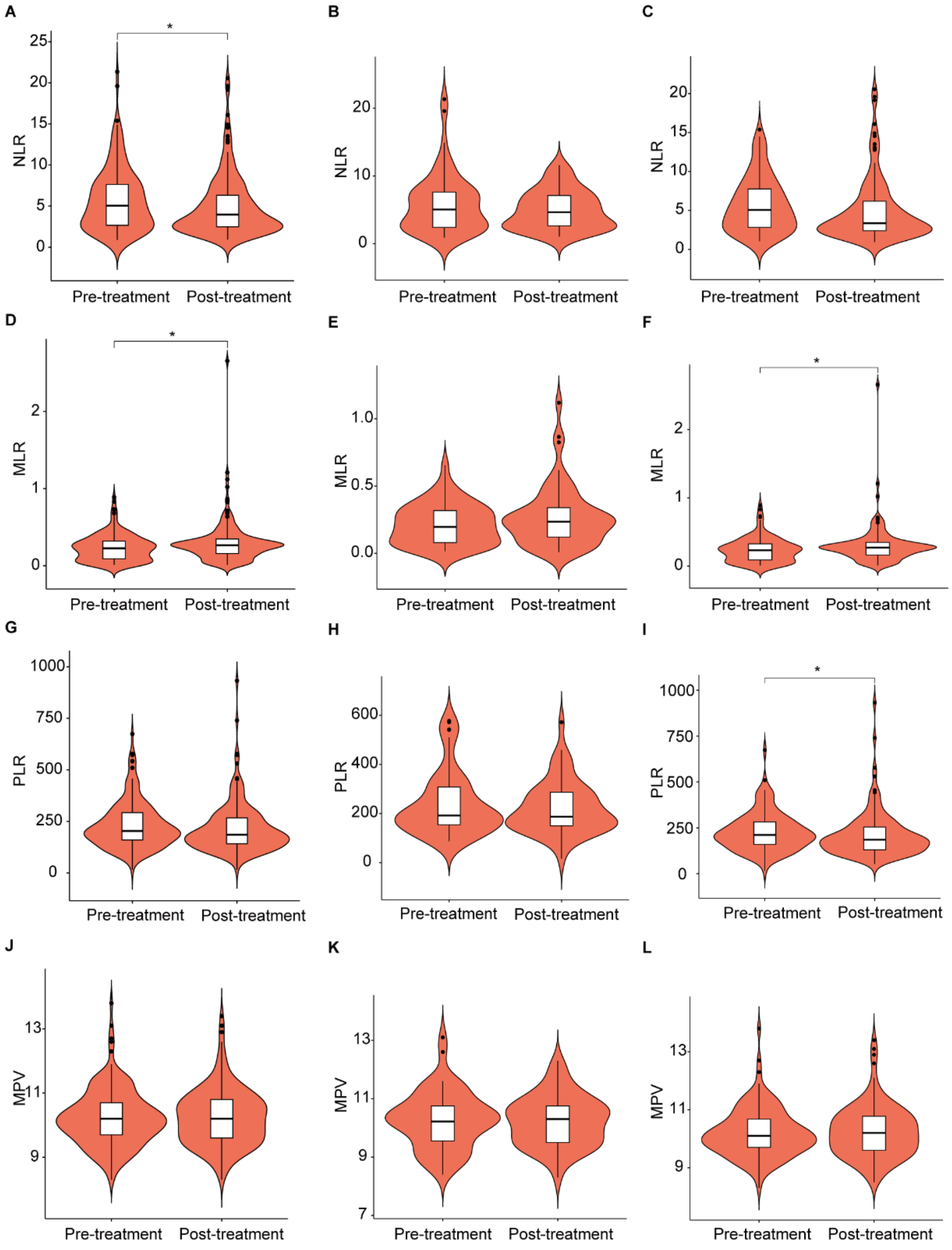

**Figure S3. Comparison of baseline NLR, MLR, PLR and MPV in corticosteroids treatment subgroups.**

Violin plots to show baseline NLR (A), MLR (B), PLR (C) and MPV (D) in the corticosteroids treatment effective and the ineffective groups.

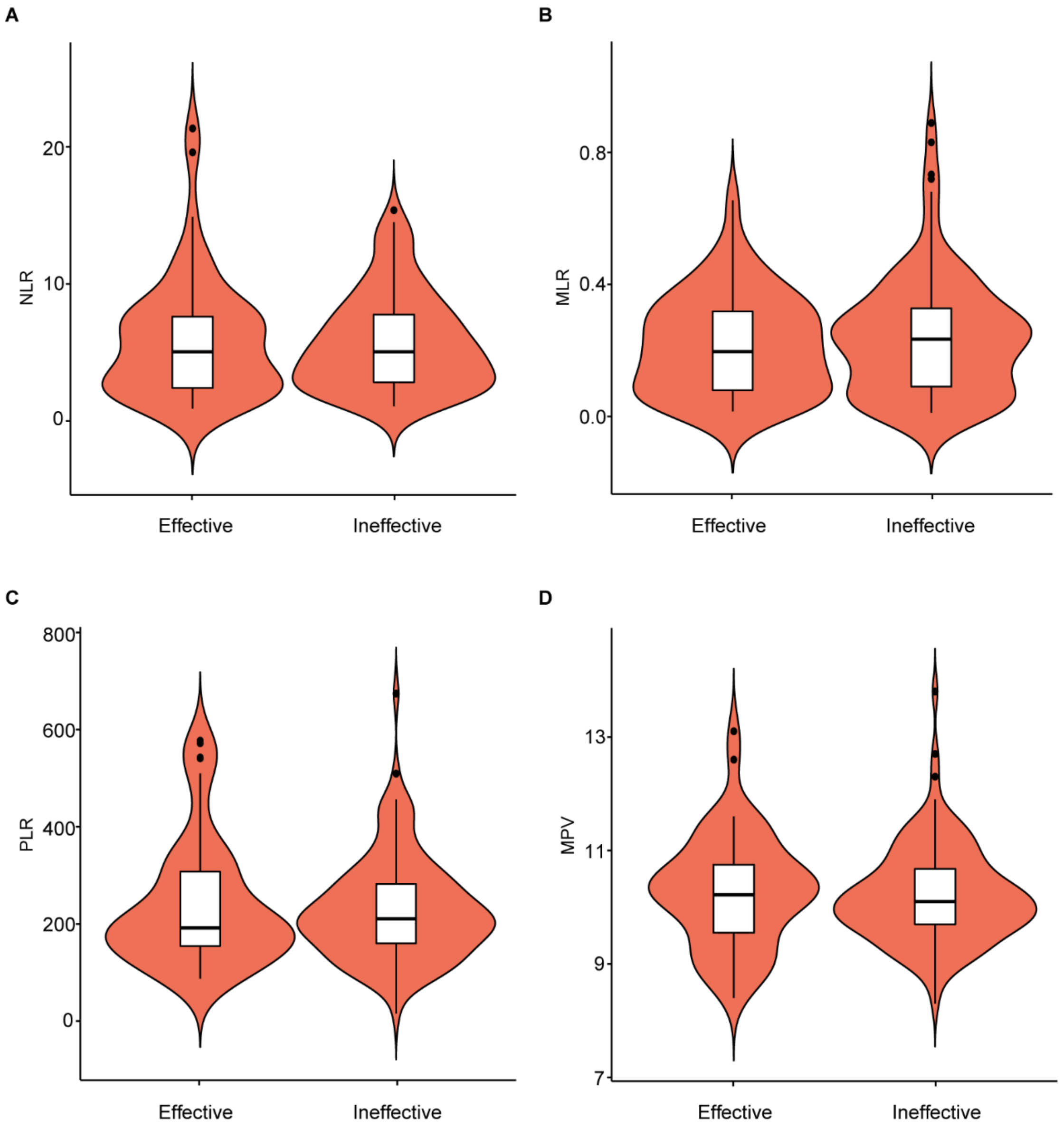

Supplement: Supplementary file 1 — Supporting Information [file CTM2-12-e583-s001.pdf]
